# Chorioangioma of Placenta: A Rare Placental Cause for Adverse Fetal Outcome

**DOI:** 10.1155/2012/913878

**Published:** 2012-06-13

**Authors:** Sreelakshmi Kodandapani, Abha Shreshta, Vani Ramkumar, Lakshmi Rao

**Affiliations:** ^1^Department of Obstetrics and Gynecology, Kasturba Medical College, Manipal University, Manipal 576104, India; ^2^Department of Pathology, Kasturba Medical College, Manipal Universty, Manipal 576104, India

## Abstract

Chorioangioma is a benign angioma of placenta arising from chorionic tissue. Large chorioangioma has unfavourable effects on both mother and fetus. We describe a case with large chorioangioma that had a poor outcome on the fetus. We also reviewed the literature on prognostic factors affecting fetal outcome.

## 1. Introduction

Chronic placental insufficiency is the commonest cause for fetal growth restriction. Rare placental causes affecting fetal outcome are partial mole, chorioangioma, and placental teratoma. Large chorioangioma has adverse effects on both mother and fetus. We report a huge chorioangioma resulting in polyhydramnios, preterm labor, and neonatal death due to congestive cardiac failure. 

## 2. Case Report

A 27-year-old third gravida, one infant death, one living child who were born vaginally presented to us at 32 weeks gestation with history of gradual abdominal distension and vague pain abdomen since one month. On examination, blood pressure was 130/80 mm Hg, abdomen was overdistended upto xiphisternum, fetal parts were not palpable, and fetal heart sounds could not be localized. She was neither diabetic nor anemic. 

**Figure 1:**
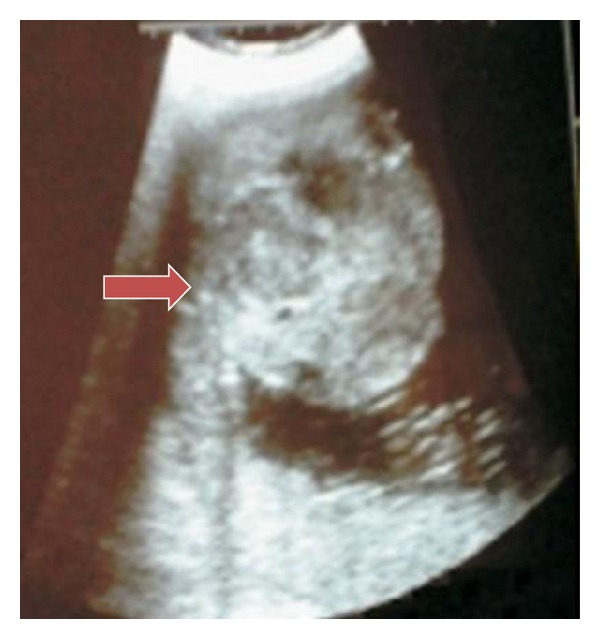
Transabdominal ultrasound showing echogenic large lobular mass suggestive of chorioangioma measuring approximately 12 × 12 cm, arrow pointing to tumor.

**Figure 2:**
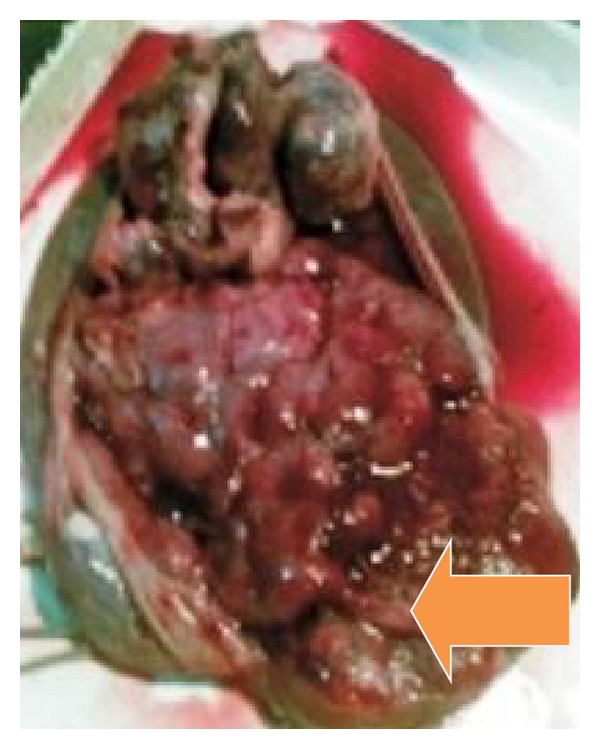
A huge highly vascular tumor suggestive of chorioangioma with placenta weighing 2 kg after delivery, arrow pointing to tumor.

**Figure 3:**
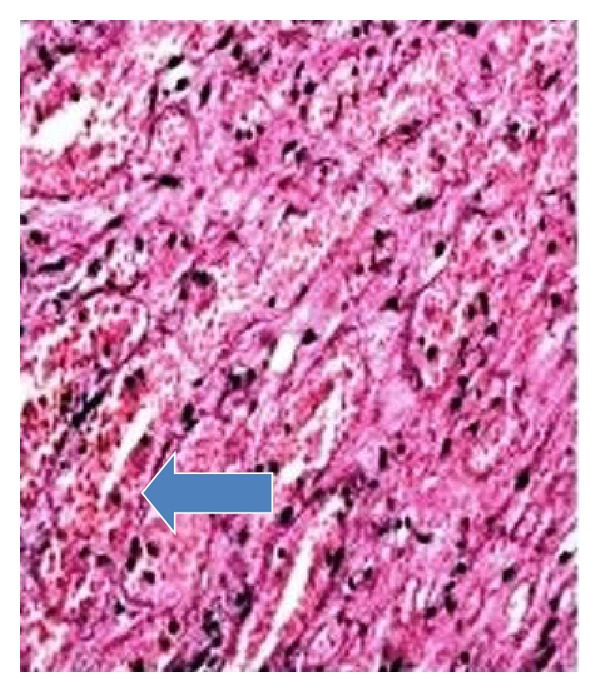
Microscopic examination of chorioangioma with lakes of blood vessels suggestive of angiomatous pattern, arrow indicating vessels.

Ultrasound showed a single live fetus corresponding to 32 weeks of gestation with polyhydramnios (AFI: 28 cm). Normal amniotic fluid index ranges from 8 to 20 cm. There were no gross structural abnormalities. Placenta was on the anterior wall upper segment, grade II. A well-defined echogenic mass measuring 11.5 cm × 12 cm different from the rest of the placenta was seen bulging on the fetal side (Figure 1). Patient went into spontaneous preterm labor and delivered female baby weighing 1.6 Kg with Apgar scores 9 and 10 at 1 and 5 minutes, respectively. Placenta weighed 2 Kg. A lobular mass measuring 12 cm × 12 cm was attached to the fetal surface of placenta with a pedicle (Figure 2). Baby died of DIC on 3rd postnatal day. Histopathology of placenta was angiomatous pattern of chorioangioma (Figure 3).

## 3. Discussion

### 3.1. Pathogenesis and Pathology

Placental chorioangioma is the most common benign tumor of the placenta. The largest retrospective study of 22000 placental examinations showed 138 chorioangioma with an incidence of 0.6% [1]. They were more seen in multiple pregnancies and in female babies. Chorioangioma is believed to arise by 16th day of fertilization, although there is no documentation of tumor in first trimester [2]. It consists of a benign angioma arising from chorionic tissue. Three histological patterns of chorioangiomas have been described by Marchetti [3] (Figure 3): angiomatous, cellular, and degenerate. The angiomatous is the most common, with numerous small areas of endothelial tissue, capillaries, and blood vessels surrounded by placental stroma.These lesions are sometimes classified as placental hamartomas rather than true neoplasia. [1]. There is no malignant potential. 

### 3.2. Clinical Features and Complications

Tumors of less than 5 cm are usually asymptomatic and unlikely to cause maternal and fetal complications. Large tumors probably act as arteriovenous shunts and cause complications. Maternal complications are *preeclampsia*, *preterm labour*, *placental abruption*, and *polyhydramnios.* Of the various reported clinical complications, the correlation of chorioangioma with hydramnios and preterm delivery is significant, latter being a sequelae of the hydramnios. *Fetal congestive heart failure* may develop because of the increased blood flow through the low resistance vascular channels in the chorioangioma acting as an arteriovenous shunt. Other associated complications are *hydrops*, *hemolytic anemia*, *congenital anomalies*, *fetal thrombocytopenia*, *cardiomegaly*, and *growth restriction.*

### 3.3. Ultrasound Diagnosis

Clarke in 1978 described the first case of chorioangioma [4]. Antenatal ultrasound was reported in 1978, and this has made diagnosis and follow up possible before delivery [2]. *This also helps in close fetal monitoring and hence timely delivery.* Gray-scale findings are well-defined complex echogenic mass different from the rest of placenta and tumor *protrudes into amniotic cavity near umbilical cord insertion.* Use of Doppler to differentiate from placental teratoma, blood clot, and leiomyoma was first demonstrated by Bromley and Benacerraff [5]. On Doppler, feeding vessel usually has same pulsatile flow as that of umbilical artery but may have arteriovenous shunt causing low resistance flow [6]. Unfortunately, we could not do Doppler in our case due to lack of facilities. Even MRI has been done in tumors that look similar to myoma in ultrasound. T2 images of MRI will be similar to hemangioma and hence diagnosis is possible [7]. 

### 3.4. Interventions

Chorioangioma with complications before fetal viability requires interventions. Various techniques with varying success rates have been tried such as serial fetal transfusions [8], fetoscopic laser coagulation of vessels supplying the tumor [9], chemosclerosis with absolute alcohol [10], and endoscopic surgical devascularization. Polyhydramnios is treated with therapeutic amniocentesis and maternal indomethacin therapy. Steroid administration for acceleration of fetal lung maturity before 34 weeks is indicated. However, our patient was an unbooked case and delivered within 48 hours of admission.

### 3.5. Prognostic Factors

Large chorioangioma associated with polyhydramnios leads to high perinatal morbidity and mortality, like in this case. Postpartum hemorrhage is a well-known complication in mother. 

### 3.6. Differential Diagnosis

Chorioangioma is often confused with placental teratoma, degenerated myoma, and blood clot. Chorioangioma is differentiated from the rest by demonstration of vascular channels similar to fetal vessels. Echo pattern of blood clot differs with time, while chorioangioma remains same. Partial mole has diffuse pattern and myoma is seen in maternal surface [2].

## 4. Conclusion

High fetal death in a case of large chorioangioma warrants institutional and timely delivery as seen in our case. Antenatal diagnosis is by ultrasound, and Doppler would have been the investigation of choice in accurate diagnosis of chorioangioma. Regular followup helps in timely diagnosis and intervention.

## References

[1] Kuhnel P (1933). Placental chorioangioma. *Acta Obstetricia et Gynecologica Scandinavica*.

[2] Bracero LA, Davidian M, Cassidy S www.thefetus.net.

[3] Marchetti AA (1939). A consideration of certain types of benign tumors of the placenta. *Surgery, Gynecology & Obstetrics*.

[4] Asokan S, Chad AK, Gard R (1978). Prenatal diagnosis of placental tumor by ultrasound. *Journal of Clinical Ultrasound*.

[5] Bromley B, Benacerraf BR (1994). Solid masses on the fetal surface of the placenta: differential diagnosis and clinical outcome. *Journal of Ultrasound in Medicine*.

[6] Sepulveda W, Aviles G, Carstens E, Corral E, Perez N (2000). Prenatal diagnosis of solid placental masses: the value of color flow imaging. *Ultrasound in Obstetrics and Gynecology*.

[7] Mochizuki T, Nishiguchi T, Ito I (1996). Case report of antenatal diagnosis of chorioangioma of the placenta: MR features. *Journal of Computer Assisted Tomography*.

[8] Zalel Y, Weisz B, Gamzu R, Schiff E, Shalmon B, Achiron R (2002). Chorioangiomas of the placenta: sonographic and doppler flow characteristics. *Journal of Ultrasound in Medicine*.

[9] Quintero RA, Reich H, Romero R, Johnson MP, Gonçalves L, Evans MI (1996). In utero endoscopic devascularization of a large chorioangioma. *Ultrasound in Obstetrics and Gynecology*.

[10] Wanapirak C, Tongsong T, Sirichotiyakul S, Chanprapaph P (2002). Alcoholization: the choice of intrauterine treatment for chorioangioma. *Journal of Obstetrics and Gynaecology Research*.

